# Superior Immunogenicity of Inactivated Whole Virus H5N1 Influenza Vaccine is Primarily Controlled by Toll-like Receptor Signalling

**DOI:** 10.1371/journal.ppat.1000138

**Published:** 2008-08-29

**Authors:** Felix Geeraedts, Nadege Goutagny, Veit Hornung, Martina Severa, Aalzen de Haan, Judith Pool, Jan Wilschut, Katherine A. Fitzgerald, Anke Huckriede

**Affiliations:** 1 Department of Medical Microbiology, Molecular Virology Section, University Medical Center Groningen and University of Groningen, Groningen, The Netherlands; 2 Department of Medicine, Division of Infectious Diseases and Immunology, University of Massachusetts Medical School, Worcester, Massachusetts, United States of America; National Institutes of Health, United States of America

## Abstract

In the case of an influenza pandemic, the current global influenza vaccine production capacity will be unable to meet the demand for billions of vaccine doses. The ongoing threat of an H5N1 pandemic therefore urges the development of highly immunogenic, dose-sparing vaccine formulations. In unprimed individuals, inactivated whole virus (WIV) vaccines are more immunogenic and induce protective antibody responses at a lower antigen dose than other formulations like split virus (SV) or subunit (SU) vaccines. The reason for this discrepancy in immunogenicity is a long-standing enigma. Here, we show that stimulation of Toll-like receptors (TLRs) of the innate immune system, in particular stimulation of TLR7, by H5N1 WIV vaccine is the prime determinant of the greater magnitude and Th1 polarization of the WIV-induced immune response, as compared to SV- or SU-induced responses. This TLR dependency largely explains the relative loss of immunogenicity in SV and SU vaccines. The natural pathogen-associated molecular pattern (PAMP) recognized by TLR7 is viral genomic ssRNA. Processing of whole virus particles into SV or SU vaccines destroys the integrity of the viral particle and leaves the viral RNA prone to degradation or involves its active removal. Our results show for a classic vaccine that the acquired immune response evoked by vaccination can be enhanced and steered by the innate immune system, which is triggered by interaction of an intrinsic vaccine component with a pattern recognition receptor (PRR). The insights presented here may be used to further improve the immune-stimulatory and dose-sparing properties of classic influenza vaccine formulations such as WIV, and will facilitate the development of new, even more powerful vaccines to face the next influenza pandemic.

## Author Summary

The rise and spread of the highly pathogenic avian H5N1 influenza virus has seriously increased the risk of a new influenza pandemic. However, the number of vaccine doses that can be produced with today's production capacity will fall short of the demand in times of a pandemic. Use of inactivated whole virus (WIV) vaccines, which are more immunogenic than split virus or subunit vaccines in an unprimed population, could contribute to a dose-sparing strategy. Yet, the mechanisms underlying the superior immunogenicity of WIV vaccine formulations are unknown. Here, we demonstrate that the viral RNA present in inactivated virus particles is crucial for the improved immunogenic properties of WIV in mice. By triggering Toll-like receptor 7 (TLR7), the viral RNA activates innate immune mechanisms that augment and determine subsequent adaptive responses. Efficient TLR7 signalling is lost in split virus and subunit vaccines with the processing steps that lead to disruption of the integrity of the virus particle and exclusion of the RNA. Our results prove for the first time to our knowledge that the immune-potentiating mechanism of a classic vaccine is based on activation of the innate immune system by one of its structural components. These findings may reflect a general principle for viral vaccines and provide a rational basis for further improvement of influenza vaccines, which are urgently needed in the face of the current H5N1 pandemic threat.

## Introduction

The first cases of human infection with highly pathogenic avian influenza (HPAI) H5N1 virus occurred in 1997 during an outbreak in Hong Kong [1]. Since then HPAI H5N1 has spread across Asia, Europe, Africa and the Pacific, and has caused a cumulative number of 338 laboratory confirmed human cases of infection, with a fatality rate of >60% [2]. Although no sustained human to human transmission has been observed yet, the threat of an imminent H5N1 pandemic requires maximum preparedness [3]. Vaccination is considered the cornerstone of protection against epidemic and pandemic influenza. However, an anticipated scarcity of the antigenic vaccine components and a narrowed time window between vaccine production and deployment puts special constraints on the vaccine formulation to be used in a pandemic situation [4],[5]. Consequently, pandemic vaccine formulations should ideally be dose sparing and uncomplicated to produce [6],[7].

Whole inactivated virus (WIV) vaccines consisting of formalin-inactivated whole virus particles were the first registered influenza vaccines licensed in 1945 in the United States [8]. However, the use of this vaccine formulation caused a relatively high incidence of adverse events, including local reactions at the site of injection and febrile illness, particularly among children [9],[10]. In the 1960 and 1970s, WIV vaccines were therefore largely replaced by less reactogenic split virus (SV) and subunit (SU) formulations [8]. SV and SU vaccines contain detergent- and/or ether-disrupted (split) virus particles or purified viral haemagglutinin (HA) and neuraminidase (NA) proteins, respectively. Apparently, disruption of whole inactivated influenza virus particles diminishes the reactogenicity of the vaccines.

In primed individuals, unadjuvanted WIV, SV, and SU vaccines in general induce similar immune responses in terms of haemagglutination inhibition (HI) titres (for a meta-analysis over 24 studies see [11]). However, in individuals that have not been exposed to the vaccine antigens before, WIV vaccines are more immunogenic than SV and SU vaccines [9],[11],[12]. Similarly, in naïve animals immunization with WIV raises stronger immune responses than immunization with SV or SU [13]–[15], especially after a single administration. In the case of an H5N1 pandemic, the majority of the population is expected to be immunologically naïve to the H5N1 subtype. In this scenario, use of WIV as basis for an optimized vaccine may be of advantage, for its immunogenic superiority seems to rely on the ability to activate unique mechanisms in the priming event of the immune response.

Thus, WIV seems to harbour an intrinsic immune-potentiating component that is lost during processing of inactivated virus particles to SV and SU vaccine formulations. In earlier experiments, we and others observed that immunization of mice with WIV vaccine results in a Th1-skewed immune response and strong antibody induction with high levels of IgG2a antibodies [14]–[16]. This response type was found irrespective of the murine genetic background or subtype of virus (either H1N1 or H3N2) and conferred protective immunity against challenge with homologous virus [15],[16]. By contrast, immunization with SU vaccine yielded responses of a Th2 phenotype with lower antibody levels mainly consisting of the IgG1 subtype, which did not lead to protection. “Empty” reconstituted viral envelopes (virosomes) resembling intact virus particles but devoid of the viral nucleocapsid elicited responses similar to those after vaccination with SU formulations [15]. This identifies the viral nucleocapsid which contains the viral genomic ssRNA as the immune-potentiating component of WIV.

In the past decade, it has become increasingly clear that the acquired immune response to microbial infection is regulated through recognition of pathogen-associated molecular patterns (PAMPs) by Toll-like receptors (TLRs) and other pattern recognition receptors of the innate immune system [17]–[20]. However, the importance of TLR signalling in immune responses to vaccines remains largely unclear. A recent study showed that TLR signalling is not important for the antibody-enhancing effect of classical vaccine adjuvants such as Complete Freund's adjuvant (CFA) [21]. Since CFA contains dried mycobacteria, and therefore mycobacterial PAMPs [17], this observation casts doubt on the importance of PAMPs and TLRs in augmenting immune responses to vaccination. Influenza viral genomic ssRNA is a natural PAMP recognized by TLR7 [22]. Here, we investigate whether PAMP recognition by TLRs, in particular recognition of viral ssRNA by TLR7, is responsible for the superior response to WIV vaccines compared to SV and SU influenza vaccine formulations.

## Results/Discussion

To analyze the role of ssRNA and other PAMPs in the response to influenza vaccines in detail, we immunized wild-type C57BL/6 mice, TLR7 knock-out mice, and MyD88/TRIF double knock-out mice with different vaccine formulations. MyD88 (myeloid differentiation factor 88) is an adaptor molecule which functions downstream of all known TLRs and IL1R family members with the exception of TLR3, which instead recruits a MyD88-related adapter molecule, TRIF (TIR domain-containing adaptor protein inducing interferon β) [17]. Consequently, a deficiency of both MyD88 and TRIF excludes signalling by all TLRs. Mice were immunized intramuscularly with β-propiolactone-inactivated H5N1 (NIBRG-14) WIV, SV, or SU vaccine. Quantitative PCR using primers specific for segment 7 of the viral genome revealed that WIV contained per vaccine dose at least 5×10^8^ copies of viral RNA, the natural ligand of TLR7. In SU or SV vaccine the amount of RNA was 500 and 5,000 times lower than in WIV, respectively. Four weeks after immunization, serum and spleen cells were collected for evaluation of humoral and cellular immune responses.

**Figure 1:**
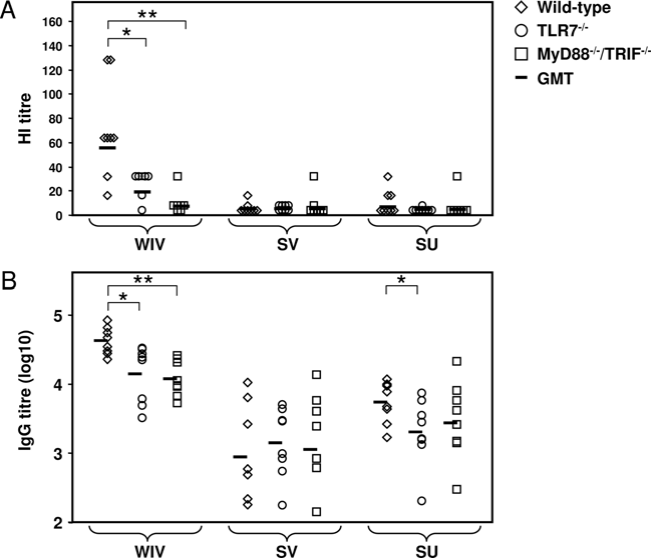
TLRs contribute to the efficacy of H5N1 WIV vaccine. Four weeks after immunization of wild-type, TLR7^−/−^, and MyD88^−/−^/TRIF^−/−^ mice with WIV, SV, or SU vaccine (5 µg HA), serum HI titres (A) and H5N1-specific IgG titres (B) were determined for the individual mice. Titres below the detection limit were assigned with half the value of the lowest detectable serum dilution, which was 8 in the HI assay and 100 in the IgG ELISA. Significant (*p*<0.05) and highly significant (*p*<0.01) differences between wild-type mice and mutant mice receiving the same vaccine are indicated by * and **, respectively. GMT indicates geometric mean titter.

Serum HI titres in WIV-immunized TLR7^−/−^ mice and MyD88^−/−^/TRIF^−/−^ mice were found to be significantly lower than in WIV-immunized wild-type mice (Figure 1A; *p* = 0.021 and *p* = 0.001, respectively). Although sera from TLR7^−/−^ mice immunized with WIV showed a higher geometric mean titre (GMT) than sera from WIV-immunized MyD88^−/−^/TRIF^−/−^ mice, this difference was not significant (*p* = 0.053). Most of the HI titres of SV- and SU-immunized wild-type mice were below detection level, precluding evaluation of the effect of the knock-out mutations on the HI responses to these vaccines.

Similar to the HI titres, virus neutralization (VN) titres of pooled serum samples from mice immunized with WIV were lower in the knock-out groups than in the wild-type group (Table 1). These results clearly show that TLR signalling is critically involved in the response to WIV immunization. Yet, in the knock-out groups, VN titres obtained after immunization with WIV were still modestly higher than those obtained after vaccination of wild-type mice with the other vaccines. This points to TLR-independent pathways contributing to the superior antibody response to WIV vaccine.

**Table 1:** TLR-dependent and -independent mechanisms contribute to virus neutralization titres induced by WIV.

Vaccine	Mouse Strain	VN Titre
	wt	640
WIV	TLR7^−/−^	80
	MyD88^−/−^/TRIF^−/−^	80
	wt	20
SV	TLR7^−/−^	20
	MyD88^−/−^/TRIF^−/−^	40
	wt	40
SU	TLR7^−/−^	20
	MyD88^−/−^/TRIF^−/−^	20

Mouse sera were collected 4 wk after immunization with different vaccine formulations and pooled per immunization group (n = 8 per group, except for SU immunized MyD88^−/−^/TRIF^−/−^ mice: n = 7) and subsequently submitted to the VN assay.

Serum titres of H5N1-specific IgG were determined by ELISA. In accordance with the HI and VN results, IgG titres were significantly decreased in WIV-immunized TLR7^−/−^ and MyD88^−/−^/TRIF^−/−^ mice compared to wild-type mice (Figure 1B; *p = *0.010 and *p = *0.001, respectively). However, like the VN titres, the IgG titres in the WIV-immunized mutant mice were still significantly higher than those induced by SV (TLR7^−/−^: *p = *0.001; MyD88^−/−^/TRIF^−/−^: *p = *0.005) or SU (TLR7^−/−^: *p = *0.005; MyD88^−/−^/TRIF^−/−^: *p = *0.021) immunization again indicating involvement of TLR-independent pathways. The relative contributions of TLR-dependent and -independent mechanisms to the superior IgG response to WIV can be estimated by comparing the difference in geometric mean titre (GMT) between WIV-immunized wild-type and MyD88/TRIF-deficient mice with the difference between WIV-immunized wild-type mice and SV- or SU-immunized wild-type mice. Using this procedure the TLR-dependent contribution was calculated to be 73% and 83% for WIV versus SV and WIV vs SU, respectively (for calculation, see Text S1). The IgG responses to SV and SU vaccine in both TLR7^−/−^ or MyD88^−/−^/TRIF^−/−^ mice did not differ from those in wild-type mice, except for the IgG response to SU in TLR7^−/−^ mice, which was slightly but significantly decreased (*p = *0.038; Figure 1B). Together with the HI and VN results, these findings demonstrate that the superior antibody response to WIV is predominantly regulated by TLRs, TLR7 in particular, while TLRs do not seem to play a prominent role in SV and SU antibody responses.

**Figure 2:**
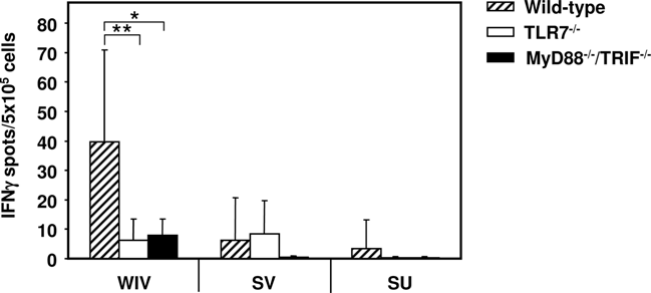
Induction of IFNγ-producing T cells by H5N1 WIV vaccine depends on TLR7 signalling. Spleen cells of wild-type mice and mutant mice immunized with WIV, SV, or SU vaccine were re-stimulated *in vitro* with SU vaccine, and numbers of IFNγ-producing cells were determined by Elispot assay. Bars represent the average values of triplicate determinations per mouse for each mouse type and immunization group (n = 8; MyD88^−/−^/TRIF^−/−^/SU, n = 7), with standard deviation. Significant (*p*<0.05) and highly significant (*p*<0.01) differences between wild-type mice and mutant mice receiving the same vaccine are indicated by * and **, respectively.

We next investigated the role of TLRs in the Th1 polarization of the response characteristically found after WIV vaccination. We first assessed numbers of IFNγ- and IL4- producing T cells (Th1 and Th2 cells, respectively) in a cytokine-specific Elispot assay, after re-stimulation of spleen cells from immunized mice with H5N1 SU vaccine. Numbers of Th1 cells were significantly decreased in WIV-immunized knock-out mice compared to wild-type mice (*p = *0.003 and *p = *0.010 for TLR7^−/−^ and MyD88^−/−^/TRIF^−/−^ mice, respectively), and matched those found in SV- and SU-immunized wild-type mice (Figure 2). No difference was found between TLR7^−/−^ and MyD88^−/−^/TRIF^−/−^ mice. Numbers of influenza-specific IL4-producing cells were extremely low in all animals for all vaccine formulations without significant differences between knock-out and wild-type mice (not shown). These data indicate that stimulation of TLR7 by ssRNA is the predominant determinant of the strong Th1-type cellular response induced by WIV.

**Figure 3:**
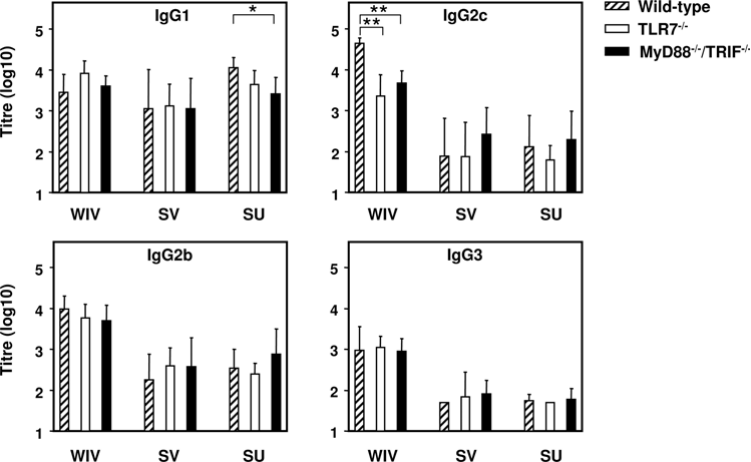
H5N1 WIV vaccine induces Th1-type antibody responses via TLR7 signalling. Serum titres of H5N1-specific IgG1 subtype (Th2-type antibody), and IgG2c, IgG2b, and IgG3 subtypes (Th1-type antibodies) were determined by ELISA. Geometric mean titres are plotted for each group of wild-type mice or mutant mice (n = 8; MyD88^−/−^/TRIF^−/−^/SU, n = 7) immunized with WIV, SV, or SU vaccine. Significant (*p*<0.05) and highly significant (*p*<0.01) differences between wild-type mice and mutant mice receiving the same vaccine are indicated by * and **, respectively.

We further determined the subtype profiles of H5N1-specific serum IgG by ELISA (Figure 3). IFNγ is known to stimulate production of IgG2a subtype antibodies by activated B cells, while IL4 stimulates IgG1 secretion [23]. In C57BL/6 mice, however, the IgG2c subtype is produced instead of IgG2a [24],[25]. Hence, a predominance of IgG2c or IgG1 is indicative of a Th1- or Th2-type response, respectively. WIV immunization of TLR7^−/−^ mice as well as MyD88^−/−^/TRIF^−/−^ mice resulted in significantly reduced IgG2c levels as compared to wild-type mice (Figure 3; *p = *0.001 for both types of knock-out mice), supporting a role for TLR7 in Th1 polarization. IgG1 was increased in WIV-immunized TLR7^−/−^ mice (*P* = 0.050), adding to the preponderance towards a Th2-type response to WIV in these mice. The average of ratios of serum IgG2c and IgG1 concentrations (determined with appropriate IgG subtype protein standards) was 17.82 (SD 8.44) for the wild-type mice immunized with WIV, compared to 0.53 (SD 0.41) for TLR7^−/−^ mice immunized with WIV. SV and SU vaccines induced predominantly IgG1 and low levels of IgG2c, consistent with a Th2-type response (Figure 3). For reasons unknown, SU vaccine induced lower IgG1 titres in both types of knock-out mice compared to the wild-type mice (TLR7^−/−^: *p = *0.050; MyD88^−/−^/TRIF^−/−^: *p = *0.014). Whether the presence of some residual RNA in SU vaccine might play a role remains to be shown.

The response characteristics of the different H5N1 vaccines in wild-type mice were well in line with those previously found for other influenza subtypes [15],[16]. This consistency is supportive of a general mechanism underlying the differences in responses to WIV, SV and SU vaccine, which operates irrespective of the virus subtype used to vaccinate.

**Figure 4:**
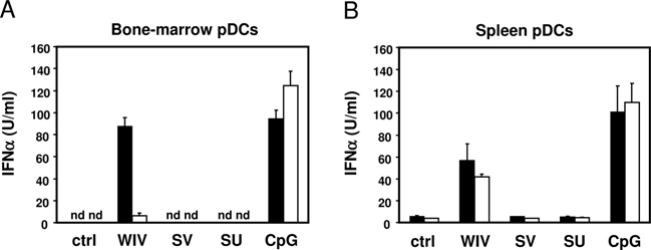
Induction of IFNα by WIV is TLR7-dependent in bone-marrow derived pDCs, but not in spleen-derived pDCs. Bone-marrow cells cultured with FLT3L (containing 20–30% pDCs) (A), or pDC-enriched spleen cell cultures (containing 62%–68% pDCs) (B) of wild-type mice (black bars) and TLR7^−/−^ mice (white bars) were incubated overnight with WIV, SV, or SU vaccine. IFNα was measured in cell supernatants by sandwich ELISA. Bars represent average values of triplicate determinations with standard deviation, and are representative of three independent experiments.

The above results demonstrate that TLR signalling plays an important role in the magnitude and Th1 skewing of the response to WIV influenza vaccines. Yet, in TLR-ko mice, WIV remained more immunogenic than SV and SU vaccines, inducing significantly higher titres of total IgG (Figure 1B) and Th1-type antibody subtypes (IgG2b, IgG2c, IgG3; Figure 3; *p*<0.05 for all comparisons). Thus, next to TLR-dependent mechanisms, a (minor) TLR-independent factor seems to contribute to the superior magnitude and Th1-skewing of the immune response to WIV. Type I interferons, including IFNα, have been shown to stimulate antibody responses and isotype switching to IgG2a when added to influenza subunit vaccine or other protein antigens [26],[27], even without the need for additional TLR stimuli. We have previously shown for an H3N2 influenza virus strain that, unlike SU vaccine, WIV vaccine efficiently induced interferon α (IFNα) production in plasmacytoid dendritic cells (pDCs) *in vitro* [15]. We therefore evaluated the induction of IFNα by the H5N1 influenza vaccine formulations used in this study and its TLR7 dependency *in vitro*. In pDCs of wild-type mice cultured from bone marrow cells (Figure 4A, black bars) or enriched from splenocytes (Figure 4B, black bars) WIV but not SV or SU induced IFNα production. In bone marrow-derived pDCs from TLR7^−/−^ mice, IFNα production upon incubation with WIV was strongly decreased as compared to wild-type DCs (Figure 4A), confirming the results of others [22]. However, spleen-derived pDCs from TLR7^−/−^ mice exposed to WIV produced similar amounts of IFNα as compared to pDCs from wt mice (Figure 4B). Thus, while in pDCs cultured from bone marrow induction of IFNα production by WIV is strictly dependent on TLR7, in pDCs enriched directly from spleen cells it is independent of TLR7. This implies that bone marrow pDCs and spleen pDCs are not completely identical. In line with this notion, bone marrow pDCs and spleen pDCs were earlier found to respond differently to HSV virus infection with respect to the TLR9 dependency of the IFNα response [28]. Our results show that WIV is indeed able to induce IFNα in a TLR7-independent way. This may also be the case in the in vivo situation, where in accordance with its well-described adjuvant functions IFNα may lead to the production of Th1 type antibodies in TLR-deficient mice [26].

Possible TLR-independent pathways activated by WIV may involve the retinoic acid-inducible gene *(RIG-I)* [29]–[32]. *RIG-I* is a cytoplasmic RNA-helicase that recognizes influenza virus by binding viral ssRNA bearing 5′-triphosphates which leads to IFNα production [33],[34]. The inactivated virus particles in WIV vaccine retained their membrane-fusion property (Text S2) and part of the viral genomes could therefore have entered the target cell cytoplasm to be sensed by *RIG-I.*

Taken together our observations show that the superior immune response to WIV, relative to that to SV or SU vaccines, is driven primarily by TLR-dependent mechanisms. Herein the presence of the viral RNA in the vaccine seems to play a crucial role. In contrast to SV and SU vaccines WIV contains substantial amounts of viral RNA. Removal of ssRNA from WIV by detergent solubilization and ultracentrifugation followed by reconstitution of the viral membrane envelopes to virosomes abolishes the capacity of the vaccine to induce production of IFNα by pDCs *in vitro* (Text S3 and Figure S1A) and type 1 immune responses *in vivo* [15]. On the other hand, ssRNA purified from WIV and condensed with polyethylenimine (PEI) did induce IFNα production in vitro (Text S3 and Figure S1B). Obviously, exposure of the viral RNA to β-propiolactone in the course of virus inactivation leaves the RNA intact to trigger TLR7-mediated signaling pathways (Figure 4), which translates into a strong and Th1-skewed antibody response to WIV in wild-type mice. In addition, the viral RNA may contribute to the TLR-independent part of the response to WIV since TLR7-independent production of IFNα could only be induced in pDCs by WIV and not by formulations (SV, SU, or reconstituted viral envelopes) which lack viral RNA (Figure 4B) [15]. These lines of evidence point to the ssRNA in WIV as the key component that enhances and steers the adaptive immune response by involvement of innate immune mechanisms.

IFNα induction in pDCs clearly discriminates WIV from SV and SU vaccines but seems to occur independent of TLR7. The fact that the immune response to WIV is predominantly dependent on TLR7 then suggests that other TLR7-mediated mechanisms, possibly involving conventional DCs and B cells, critically contribute to the immune reaction. Recently, an *in vitro* study on B cells showed that TLR7 stimulation or CD40-CD40L binding by itself triggers IgG1 antibody production, but when simultaneously present induce proliferation and a switch to IgG2a production [25]. Additional stimulation of IFNα/β receptors on the same cells further drives the production of IgG2a at the expense of IgG1 antibodies [25]. Although this model might represent an over-simplification of the *in vivo* situation, it is in line with our data. The different scenarios encountered upon immunization of wild-type and mutant mice with WIV, SV, or SU are summarized in Table 2. WIV provides the ssRNA for direct triggering of TLR7 in B cells as well as the CD40 ligand for CD40 stimulation on B cells through strong T helper cell induction, which was shown also to depend on TLR7 signalling. Together with IFNα produced by TLR7-mediated and/or TLR7-independent mechanisms, these signals will lead to the enhanced and strongly polarized Th1-type antibody responses characteristic for WIV. In the absence of TLR7, WIV-induced IFNα can still stimulate moderate production of Th1 type antibodies and increase the total IgG. In contrast, SV and SU vaccines are poor inducers of T helper cells and IFNα, and cannot stimulate B cells directly via TLR7. Consequently, SV and SU vaccines induce lower and more Th2-polarized antibody responses.

**Table 2:** Putative vaccine effects contributing to different adaptive immune responses based on the model proposed by Heer et al. [25].

Vaccine	Mouse Strain	Direct Vaccine Effects			Result	
		IFNα Production	Th Cell Induction	TLR7-Mediated B-Cell Stimulation	Antibody Response	Phenotype
WIV	wt	+	++	+	+++	Th1
	TLR7^−/−^	+	+	−	++	Th1/Th2
SV/SU	wt	−	+	−	+	Th2
	TLR7^−/−^	−	+	−	+	Th2

Differences in responses induced in either wild-type or TLR7-deficient mice by WIV and SV or SU vaccine are given semiquantitatively for each of the indicated facets of the innate or adaptive response.

Our data provide mechanisms which explain the superiority of WIV vaccine to prime HA-specific immune responses in mice. Whether similar mechanisms are operational in humans and contribute to the stronger immunogenicity of WIV compared to SV or SU in unprimed individuals remains to be elucidated. Despite the favourable immunogenic properties of WIV, recent clinical trials performed in the context of pandemic vaccine development show that even with WIV at least two immunizations with a substantial amount of antigen (15–30 µg) and/or the addition of adjuvants will probably be required to achieve immune responses that comply with the CPMP criteria. If TLRs are involved in the priming of humans with WIV, their role during recall responses may be less critical, given the fact that in general WIV, SU, and SV induce similar HI titres in primed populations [11]. Use of WIV derived from wild-type virus instead of recombinant vaccine strains resulted in good antibody titres even without the addition of adjuvants and might thus be an option to obtain satisfying immune responses [35]. Evaluation of adjuvants in combination with WIV in clinical trials is so far restricted to aluminium salts. However, where adjuvanted and non-adjuvanted WIV were compared side-by-side, effects of this Th2 adjuvant on vaccine efficacy were absent, poor, or inconsistent [36]. So, better adjuvants have to be found that work synergistically with WIV in order to exploit the full potential of intact inactivated virus particles as vaccines.

In conclusion, our data reveal, for the first time to our knowledge, that TLRs play an eminent role in the immune responses to a classic influenza vaccine. Of the three influenza vaccine formulations studied here, only WIV efficiently triggered TLR7-mediated mechanisms leading to superior immune responses. Processing of inactivated whole virus particles into SV or SU eliminates the immuno-potentiating effect of the viral ssRNA, the primary PAMP in WIV vaccine, and results in a loss of quantity and shift in the quality of the immune response. Thus, TLR-dependent mechanisms appear to form the basis for WIV's antigen-sparing quality and hence its recognized strong potential as a pandemic vaccine candidate [7],[12]. Optimizing TLR7-signalling by rational vaccine design may produce even more potent vaccines, which are urgently needed in the face of the current influenza pandemic threat.

## Methods

### Vaccines and reagents

H5N1 virus (NIBRG-14, a 2∶6 recombinant of A/Vietnam/1194/2004 [H5N1] and A/PR/8/34 [H1N1] virus produced by reverse genetics technology) was provided by the National Institute for Biological Standards and Controls (NIBSC; Potters Bar, UK), propagated on embryonated chicken eggs, inactivated with 0.1% β-propiolactone to obtain WIV, and processed into split virus vaccine or subunit vaccine according to standard procedures [37],[38]. The haemagglutinin protein concentration in the vaccines was determined by single radial immunodiffusion (SRID) [39]. Endotoxin levels in all vaccines met the requirements of the European Pharmacopoeia standard. (If, nevertheless, contamination of endotoxin [signalling via TLR4] would have played an important role we should have observed substantial differences in the response between TLR7-deficient mice [capable of signalling via TLR4)]and MyD88/TRIF-deficient mice [deficient in all TLR-derived signalling]. However, such differences were not found for any of the vaccines.) CpG DNA (ODN D19) was purchased from Eurogentec (Seraing, Belgium).

### Mice and vaccination

For immunization experiments, C57BL/6, TLR7^−/−^ and MyD88^−/−^/TRIF^−/−^ mice (generated from MyD88^−/−^ mice [40] and TRIF^−/−^ mice [41]) were bred at the University of Massachusetts Medical School (Worcester, MA). For *in vitro* studies, 10- to 12-week-old female C57BL/6 mice were purchased from Harlan Netherlands B.V. (Zeist, The Netherlands), and TLR7^−/−^ mice (a gift from S. Akira and C. Reis e Sousa) were bred at the University Medical Center Groningen. All experiments were conducted with approval of the local Institutional Animal Care and Use Committees. Mouse groups were matched for sex and age. Groups (n = 6–8) of C57BL/6, TLR7^−/−^, and MyD88^−/−^/TRIF^−/−^ mice were intramuscularly injected with 50 µl of PBS in each calf muscle containing a total of 5 µg haemagglutinin protein per mouse of either WIV, SV, or SU vaccine formulation or no vaccine as a control. At 28 days after immunization, sera and spleens were collected for evaluation.

### Quantitative PCR

Relative viral RNA content of the different vaccines was determined using a two-step real-time RT-PCR assay amplifying a 193-bp fragment within the M1 gene of influenza A viruses. For this purpose RNA was extracted from WIV, SV, or SU (5 µg HA) with the QIAamp viral RNA Mini Kit (QIAGEN, Venlo, The Netherlands), cDNA synthesis was performed on 5 µl of viral RNA (one-tenth of the final elution volume) using the Verso cDNA kit from ABgene (Westburg, Leusden, The Netherlands), and 1 µM UNI12 primer (5′-AGCAAAAGCAGG-3′, corresponding to viral noncoding nucleotides 1 to 12 [42]). Real-time PCR was performed with 200 nM M1-FOR primer (5′-CCTGGTATGTGCAACCTGTG-3′) and M1-REV primer (5′-AGCCTGACTAGCAACCTCCA-3′); purchased from Eurogentec, and the Absolute QPCR SYBR Green Mix (ABgene). Amplification was performed on a StepOne apparatus (Applied Biosystems), and consisted of 15 min initial activation at 95°C, followed by 40 thermal cycles of 15 sec at 95°C and 60 sec at 60°C. In each experiment, a standard curve (R^2^>0.99 within the range of 1×10^2^ to 1×10^9^ copies per reaction) was drawn to convert the respective cycle threshold (C_t_) values into the number of viral genome copies. This standard consisted of a pCR2.1-TOPO plasmid construct in which was cloned a 473-bp sequence of influenza A/Puerto Rico/8/34 segment 7.

### Haemagglutination inhibition assay

The HI assay was performed as described before [15]. Briefly, heat-inactivated mouse serum was absorbed to 3 volumes 25% kaolin/PBS (Sigma-Aldrich, Inc., St. Louis, MO), 20 min at room temperature (RT). After centrifugation, 50 µl of supernatant was serially diluted two-fold in a round-bottom microtitre plate (Costar, Corning Inc., Corning, NY), in duplicate. Subsequently, 50 µl PBS was added containing 2 HAU of H5N1 (NIBRG-14) virus and incubated for 40 min at RT. We used 2 HAU of virus instead of the standard 4 HAU to increase the sensitivity of the assay. Finally, 50 µl of 1% guinea pig erythrocytes (Harlan) in PBS was added to each well and HI titres were determined after 2 h incubation at room temperature. HI titres are given as the reciprocal of the highest serum dilution producing complete inhibition of haemagglutination.

### Virus-neutralization assay

The levels of virus-neutralizing (VN) serum antibodies were determined with a VN assay [15],[43]. The VN titre was defined as the reciprocal of the highest serum dilution capable of inhibiting 200 TCID50 of H5N1 vaccine strain virus (NIBRG-14) from infecting Madin-Darby canine kidney cell monolayers in a microtiter plate. Infection was measured by an ELISA on intracellularly produced viral NP protein. Inhibition of infection by simultaneous incubation with mouse serum was established if the ELISA absorbance value (A_492_) measured was below the cut-off value, determined by the equation: [(average A_492_ of the positive controls (infected cells) minus average A_492_ of the negative controls (non infected cells)) divided by 2] plus the average A_492_ of the negative controls. Serum samples were tested in quadruplicate.

### Isotype ELISA

Microtitre plates (Greiner, Alphen a/d Rijn, The Netherlands) were coated with 0.2 µg influenza H5N1 (NIBRG-14) subunit vaccine per well in 100 µl coating buffer, overnight. After blocking with 2% milk in coating buffer for 45 min, 100 µl of two-fold serial dilutions of serum samples in 0.05%Tween 20/PBS (PBS/T) were applied to the wells and incubated for 1.5 h, in duplicate. Subsequently, 100 µl of horseradish peroxidase-conjugated goat anti-mouse IgG-isotype antibody (Southern Biotech, Birmingham, Alabama) was applied for 1 h. All incubations were performed at 37°C. Staining was performed using o-phenylene-diamine (OPD) (Eastman Kodak Company) and absorbance was read at 492 nm (A_492_) with an ELISA reader (Bio-tek Instruments, Inc.). After subtraction of background levels, serum dilutions yielding an OD of 0.2 were calculated using linear regression, of which the reciprocal of the average of the duplicates represents the titre.

### IFNγ and IL4 Elispot assays

This assay was performed as described previously [15]. In short, erythrocyte-depleted splenocytes were seeded at a concentration of 5×10^5^ cells in 100 µl medium per well, in triplicate in a microtitre plate (Greiner), which was pre-coated with anti-IFNγ or anti-IL4 capture antibody (Pharmingen, San Diego, CA) and blocked with 4% BSA/PBS (Sigma-Aldrich). Cells were stimulated with 1 µg H5N1 (NIBRG-14) subunit vaccine per well, overnight in a humidified CO_2_ incubator at 37°C. Cells were lysed with 100 µl of H_2_O per well and plates were washed extensively, after which 100 µl of biotinylated anti-IFNγ or anti-IL4 (Pharmingen) in 2% BSA/PBS was added 1 h at 37°C. Subsequently, the plates were incubated with 100 µl of alkaline phosphatase conjugated streptavidin (Pharmingen) in 2% BSA/PBS for 1 h at 37°C, spots were visualized with 5-bromo-4-chloro-3-indolylphosphate (Sigma-Aldrich) substrate immobilized in solidified agarose. Plates were scanned and spots were counted manually.

### Plasmacytoid dendritic cells

Plasmacytoid DCs were generated from bone marrow cells of C57BL/6 or TLR7^−/−^ mice by seeding 1–2×10^6^ bone marrow cells per well of a 24-well plate and culturing the cells for one week in Iscove's Modified Dulbecoo's Medium (IMDM) with 10% FCS and 100 ng/ml FLT3L (R&D Systems, Abingdon, UK) [22].

Single splenocyte suspensions were produced by collagenase D (Roche Diagnostics GmbH, Germany) treatment of the spleens, and spleen cell populations enriched for plasmacytoid DCs (pDCs) were obtained after magnetically labelling of pDCs with anti-mPDCA-1 antibody conjugated MicroBeads (Miltenyi Biotech GmbH, Germany) and separation over a MACS Column (Miltenyi), according to the manufacturers protocol. Percentages of pDCs in the positively selected population were determined by FACS analysis using anti-mPDCA-1-PE antibody (Miltenyi) and anti-CD11c-FITC (GeneTec Inc., Canada). Cell suspensions containing 1–2×10^5^ pDCs in 100 µl were seeded in a microtitre plate and stimulated in triplicate with an equal volume containing 1.0 µg HA of either WIV, SV, or SU vaccine, or 1.0 nmol CpG DNA. After 20 h of incubation in a humidified CO_2_ incubator at 37°C, supernatants were collected and subjected to the IFNα ELISA.

### IFNα ELISA

IFNα detection in cell-culture supernatants was performed using a sandwich ELISA as described previously [15]. IFNα concentrations were calculated from a recombinant IFNα (HyCult, Biotechnology, Uden, The Netherlands) standard curve performed in quadruplicate using linear regression, and expressed in units per ml.

### Statistics

Statistical analysis on HI titres, antibody titres, and Elispot counts was performed with SPSS (SPSS 1202 Inc., Chicago, IL) using the Mann-Whitney *U* test with a CI of 95%. All *p* values are two-tailed. Statistical significance was defined as *p*<0.05.

## Supporting Information

Figure S1 Effect of viral RNA.
(0.84 MB TIF)

Text S1 Relative contribution of TLR-dependent and -independent mechanisms.
(0.02 MB DOC)

Text S2 Fusion activity of vaccines.
(0.02 MB DOC)

Text S3 Viral RNA from WIV stimulates TLR7.
(0.03 MB DOC)
