# Supplementary figures and images for: Superior Immunogenicity of Inactivated Whole Virus H5N1 Influenza Vaccine is Primarily Controlled by Toll-like Receptor Signalling
Source: PLoS Pathog. 2008 Aug 29;4(8):e1000138. doi: 10.1371/journal.ppat.1000138 (PMC2516931; doi:10.1371/journal.ppat.1000138)

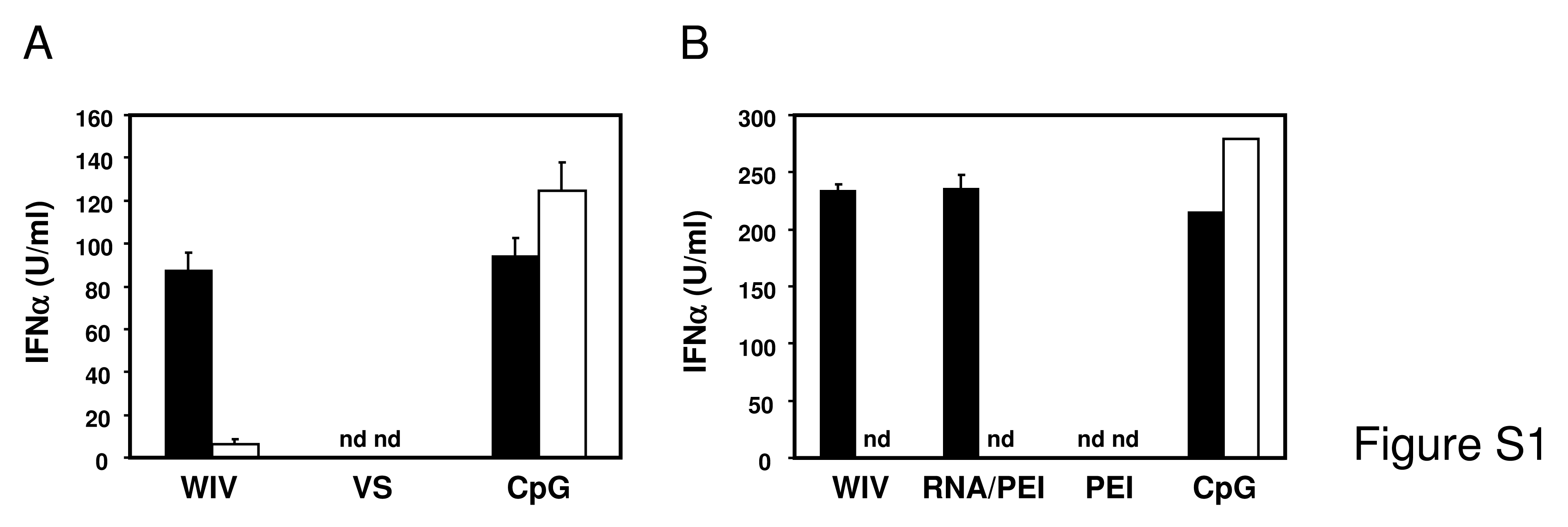

Supplement: Figure S1 — Effect of viral RNA. (0.84 MB TIF) [file ppat.1000138.s001.tif]
