# Supplementary material for: Superior Immunogenicity of Inactivated Whole Virus H5N1 Influenza Vaccine is Primarily Controlled by Toll-like Receptor Signalling
Source: PLoS Pathog. 2008 Aug 29;4(8):e1000138. doi: 10.1371/journal.ppat.1000138 (PMC2516931; doi:10.1371/journal.ppat.1000138)
Supplement: Text S1 — Relative contribution of TLR-dependent and -independent mechanisms. (0.02 MB DOC) [file ppat.1000138.s002.doc]

**Supporting Information S1. Calculation of relative contributions of TLR-dependent and -independent mechanisms to WIV’s superior IgG response.**

The contribution of TLR-dependent and -independent mechanisms to the IgG response was derived using the equation: {(x-y)/(x-z)}*100% , where x is the GMT of WIV immunized wild-type mice (=42,105), y is the GMT of WIV-immunized MyD88^-/-^/TRIF^-/-^ mice (=11,889) and z is the GMT of SV-immunized wild-type mice (=876) or, when compared to SU vaccine, the GMT of SU-immunized wild-type mice (=5,481).
