# Supplementary material for: Superior Immunogenicity of Inactivated Whole Virus H5N1 Influenza Vaccine is Primarily Controlled by Toll-like Receptor Signalling
Source: PLoS Pathog. 2008 Aug 29;4(8):e1000138. doi: 10.1371/journal.ppat.1000138 (PMC2516931; doi:10.1371/journal.ppat.1000138)
Supplement: Text S2 — Fusion activity of vaccines. (0.02 MB DOC) [file ppat.1000138.s003.doc]

**Supporting information S2. H5N1 virus inactivated with β-propiolactone is fusion-active.**

Fusion activity of H5N1 virus (NIBRG-14), inactivated with 0.1% β-propiolactone, was evaluated with a haemolysis assay and a fluorescence membrane fusion assay using WIV labelled with octadecyl Rhodamine B (R18), as described previously (Stegmann T, et al. (1993) Biochemistry 32: 11330-11337). The experimental haemolysis of human erythrocytes by WIV, as a percentage of maximal haemolysis by water treatment of the erythrocytes, was 45.2% at pH 5.5. Comparatively, haemolysis values for split virus and subunit in the same test were 0.3% and 0.5% respectively. Fusion activity, measured with the R18 assay, ranged between 25% and 30% at pH 5.5 for WIV.
