# Supplementary material for: Superior Immunogenicity of Inactivated Whole Virus H5N1 Influenza Vaccine is Primarily Controlled by Toll-like Receptor Signalling
Source: PLoS Pathog. 2008 Aug 29;4(8):e1000138. doi: 10.1371/journal.ppat.1000138 (PMC2516931; doi:10.1371/journal.ppat.1000138)
Supplement: Text S3 — Viral RNA from WIV stimulates TLR7. (0.03 MB DOC) [file ppat.1000138.s004.doc]

**Supporting Information S3. Viral RNA from β-propiolactone-inactivated WIV vaccine stimulates TLR7.**

Virosomes (VS) were prepared from β-propiolactone-inactivated H5N1 virus (NIBRG-14), by solubilization of the viral membrane, followed by removal of the nucleocapsid by ultracentrifugation, and subsequent reconstitution of the viral membrane envelope, as described previously (De Jonge J, et al. (2006) Biochimica et Biophysica Acta 1758: 527-536). Viral RNA was isolated from H5N1 WIV preparations using a RNeasy Kit (QIAGEN, Venlo, The Netherlands) and condensed with polyethylenimine (PEI, Sigma-Aldrich, Inc., St. Louis, MO), as described elsewhere (Diebold SS, et al. (2004) [Science](javascript:AL_get(this, 'jour', 'Science.');) 303: 1529-1531). Stimulation experiments were performed as described in the Methods section of the manuscript. In short, bone-marrow derived pDC cultures were stimulated with WIV or VS in a concentration of 5 μg HA per ml, or RNA/PEI complexes in a concentration of 1 μg RNA per ml, or PEI alone, or CpG DNA. The IFNα response is shown in Figure S1. Virosomes (VS) which are virus-like particles devoid of RNA do not induce detectable (nd) IFNα production in pDCs from wild-type mice (black bars) or TLR7^-/-^ mice (white bars) (Fig. S1A). In contrast RNA/PEI complexes induce IFNα production in pDCs from wild-type mice (black bars) but not from TLR7^-/-^ mice (white bars) (Fig. S1B). These experiments confirm that it is the viral genomic RNA in WIV that activates TLR7-mediated pathways, as was shown by others (Diebold SS, et al. (2004) [Science](javascript:AL_get(this, 'jour', 'Science.');) 303: 1529-1531), and that the inactivation procedure with β-propiolactone has no effect on the ability of the viral genome to stimulate TLR7.
